# NSUN2‑mediated epitranscriptomic and ubiquitin modulation of Nipah virus matrix protein reveals a dual-targeting antiviral strategy

**DOI:** 10.1093/procel/pwag003

**Published:** 2026-02-12

**Authors:** Haojie Hao, Zhen Chen, Fang Zhang, Yanling Huang, Fuyu Luo, Li Zuo, Ting Luo, Xiaoxue Wang, Caiyun Shang, Chao Shan, Haibin Liu, Xueyan Zhang, Zhiming Yuan, Fang Huang, Wuxiang Guan

**Affiliations:** Center for Emerging Infectious Diseases, Wuhan Institute of Virology, Chinese Academy of Sciences, Wuhan 430071, China; Center for Emerging Infectious Diseases, Wuhan Institute of Virology, Chinese Academy of Sciences, Wuhan 430071, China; Hubei JiangXia Laboratory, Wuhan 430200, China; Center for Emerging Infectious Diseases, Wuhan Institute of Virology, Chinese Academy of Sciences, Wuhan 430071, China; Center for Emerging Infectious Diseases, Wuhan Institute of Virology, Chinese Academy of Sciences, Wuhan 430071, China; Center for Emerging Infectious Diseases, Wuhan Institute of Virology, Chinese Academy of Sciences, Wuhan 430071, China; Center for Emerging Infectious Diseases, Wuhan Institute of Virology, Chinese Academy of Sciences, Wuhan 430071, China; College of Pharmacy, Hubei University of Chinese Medicine, Wuhan 430065, China; College of Pharmacy, Hubei University of Chinese Medicine, Wuhan 430065, China; Center for Emerging Infectious Diseases, Wuhan Institute of Virology, Chinese Academy of Sciences, Wuhan 430071, China; Center for Emerging Infectious Diseases, Wuhan Institute of Virology, Chinese Academy of Sciences, Wuhan 430071, China; Center for Emerging Infectious Diseases, Wuhan Institute of Virology, Chinese Academy of Sciences, Wuhan 430071, China; Center for Emerging Infectious Diseases, Wuhan Institute of Virology, Chinese Academy of Sciences, Wuhan 430071, China; Hubei JiangXia Laboratory, Wuhan 430200, China; Center for Emerging Infectious Diseases, Wuhan Institute of Virology, Chinese Academy of Sciences, Wuhan 430071, China

**Keywords:** Nipah virus (NiV), NSUN2, matrix (M) protein, 5-methylcytosine (m^5^C), ubiquitination, antiviral strategy

## Abstract

Nipah virus (NiV) poses a significant public health threat due to its high mortality rate and the absence of approved treatments. Nonetheless, the host–virus interactions underlying its pathogenesis remain poorly understood. Here, we identified the 5-methylcytosine (m^5^C) methyltransferase NSUN2 as a critical host factor hijacked by NiV to facilitate replication via dual mechanisms. The viral matrix (M) protein stabilizes NSUN2 by inhibiting its proteasomal degradation. In turn, NSUN2 catalyzes m^5^C deposition on NiV RNAs, enhancing M RNA stability and protein expression. Simultaneously, NSUN2’s noncatalytic domain engages GNB2 as an adaptor to facilitate the recruitment of the E3 ubiquitin ligase TRIM28 to M, promoting M ubiquitination and consequent nuclear export for virion assembly. Targeting both pathways using the proteasome inhibitor carfilzomib and the m^5^C inhibitor MY-1B suppressed NiV replication *in vitro* and in hamsters. Our findings uncover a dual epigenetic–posttranslational regulatory axis exploited by NiV and present a promising combinatorial therapeutic approach.

## Introduction

Bat-borne Nipah virus (NiV) is an emerging and highly contagious zoonotic pathogen that causes acute, frequently fatal encephalitis in humans, with mortality rate ranging from 40% to 75% (Bhattacharya et al., 2020; Chua et al., 2000; Conroy, 2023; Lo and Rota, 2008). In 2018, the World Health Organization included NiV in its Blueprint for Research and Development, recognizing it as a priority disease “capable of causing serious international outbreaks” (Mehand et al., 2018). First identified in Malaysia in 1998, NiV has since been associated with outbreaks in Singapore, India, and Bangladesh (Conroy, 2023; Eaton et al., 2006; Field et al., 2001). However, owing to strict research restrictions, including the requirement for a biosafety level 4 laboratory, the molecular mechanisms underlying NiV replication and pathogenesis remain poorly understood, and no effective vaccines and antiviral treatments have been developed.

NiV, a member of the *Paramyxoviridae* family, is classified into two genetic lineages: the Malaysian (NiV-MY) and Bengal (NiV-BD) strains (Devnath et al., 2022; Harcourt et al., 2005; Reynes et al., 2005; Wacharapluesadee et al., 2005). The virus has a single-stranded, negative-sense RNA genome that is transcribed by the RNA polymerase complex into eight mRNAs (Bose et al., 2014; Chang and Dutch, 2012; Harcourt et al., 2000; Navaratnarajah et al., 2020; Wang et al., 2022). These mRNAs encode six structural proteins, including nucleocapsid (N), phosphoprotein (P), RNA polymerase (L), matrix protein (M), fusion protein (F), and glycoprotein (G), alongside three nonstructural proteins (V, W, and C) (Ciancanelli et al., 2009; Devnath et al., 2022; Harcourt et al., 2000; Rodriguez and Horvath, 2004). Among these, the M protein is a key regulator of the NiV life cycle, playing critical roles in viral replication, immune evasion, assembly, and budding (Bharaj et al., 2016). Ubiquitination facilitates M protein transport out of the nucleus, thereby promoting viral budding (Jin et al., 2025; Pentecost et al., 2015; Y.E. Wang et al., 2010). Despite its critical roles, the host factors that interact with M and regulatory mechanisms governing its activity, particularly its ubiquitination dynamics, remain largely unknown.

Recent studies highlight the critical role of RNA modifications, such as N6-methyladenosine (m^6^A), 5-methylcytosine (m^5^C), N4-acetylcytosine (ac^4^C), and pseudouridine, in regulating viral replication and host–virus interactions. Among these, m^5^C is one of the most extensively studied. Although the roles of m^6^A in viral replications are well characterized, the mechanisms by which m^5^C regulates viral replication remain largely unexplored. The m^5^C modification is mainly catalyzed by the methyltransferase NSUN2, which regulates RNA stability, export, splicing, and translation (Zou et al., 2024). In host cells, NSUN2 is fundamentally involved in regulating biological processes, including cancer progression, neural differentiation, and stem cell renewal (Abbasi-Moheb et al., 2012; Hu et al., 2021; Phalke et al., 2009). In viruses, NSUN2-mediated RNA m^5^C modification plays diverse regulatory roles: it enhances human immunodeficiency virus type 1 replication by improving translation efficiency and modulating RNA splicing (Courtney et al., 2019b), promotes murine leukemia virus replication through increased gene expression (Courtney et al., 2019a), yet inhibits SARS-CoV-2 by reducing viral RNA stability (Wang et al., 2024). Flaviviruses, including hepatitis C virus, dengue virus, and Zika virus, are regulated by NSUN2 (Fischer et al., 2022; Hagist et al., 2009; Ruggieri et al., 2021; Wang et al., 2023; Wnuk et al., 2020). Among DNA viruses, it facilitates Epstein–Barr virus immune evasion via m^5^C modification of EBER1 while exhibiting complex effects on hepatitis B virus (HBV) replication (Ding et al., 2024; Feng et al., 2023; Henry et al., 2020). In addition, NSUN2 suppresses immune responses triggered by various viral infections (Zhang et al., 2022). Although NSUN2-mediated m^5^C modifications in positive-stranded RNA viruses are well characterized, their role in negative-stranded RNA viruses, such as NiV, remains largely unexplored. Concurrently, NSUN2 knockdown does not significantly alter the m^5^C methylation of viral RNAs but reduces the replication and gene expression of several viruses, including respiratory syncytial virus, vesicular stomatitis virus, human metapneumovirus, Sendai virus, and herpes simplex virus (Xiong et al., 2024; Zhang et al., 2022), indicating that NSUN2 may regulate viral replication through mechanisms beyond m^5^C modification. However, these potential functions remain poorly understood.

Currently, no clinically approved drugs or effective intervention strategies are available for treating NiV. However, developing anti-NiV therapeutics remains challenging, owing to the high pathogenicity of the virus, which complicates treatment, and its long incubation period (4–14 days), which hinders early detection. Several potential antiviral agents, including remdesivir (de Wit et al., 2023; Lo et al., 2019), favipiravir (T-705) (Dawes et al., 2018), 4′-azidocytidine (R1479) (Liu et al., 2025), monoclonal antibodies NiV41, NiV42 (Chen et al., 2024), m102.4 (Bossart et al., 2009; Playford et al., 2020), and nanobodies N425 (Wang et al., 2024), have been developed. However, their therapeutic potential requires further validation through additional studies. Thus, identifying new antiviral targets along with repurposing existing drugs to inhibit them could help overcome these significant challenges of NiV prevention. Additionally, research on combination therapies remains limited, as most studies focus on single-drug treatments, restricting the development of comprehensive therapeutic strategies. The NiV M protein is crucial for viral assembly and immune evasion, and its disruption halts viral spread. Characterizing M and its associated host factors could provide valuable insights for developing new intervention strategies against NiV.

Therefore, the present study aimed to investigate the crucial role of M protein–associated host factor NSUN2 in NiV replication and its potential as a therapeutic target. Two distinct mechanisms by which NSUN2 regulates M function via both m^5^C-dependent and m^5^C-independent pathways were elucidated. Our findings could provide new insights into the molecular interactions between NiV and host factors, highlighting potential therapeutic targets for inhibiting NiV replication.

## Results

### NiV M protein interacts with NSUN2 and enhances its expression

**Figure 1.**
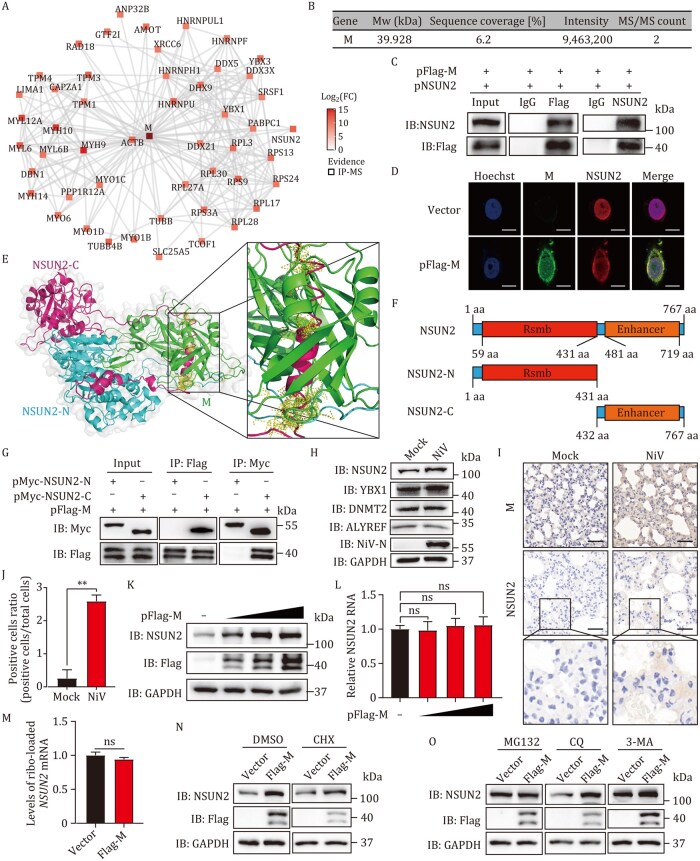
**M interacts with NSUN2 and influences its expression**. (A) Protein interaction network. Flag-M was overexpressed in HEK293T cells, followed by IP with a Flag antibody, with IgG IP serving as a control. MS was conducted to identify interacting proteins. The interaction network was constructed by integrating IP-MS data with known interactions from the STRING database. Nodes represent proteins, and edges indicate interactions. Nodes are annotated to indicate the fold change between experimental and control samples based on IP-MS data. (B) MS analysis of NiV proteins. Vero cells were infected with NiV, followed by IP using anti-NSUN2 antibodies or control IgG. The resulting protein complexes were then analyzed by MS. (C) HEK293T cells were co-transfected with pNSUN2 and pFlag-M, and co-IP was performed using indicated antibodies. (D) Confocal microscopy images of Vero cells. Cells transfected with either vector or pFlag-M were stained with anti-Flag and anti-NSUN2 antibodies. Nuclei were labeled with Hoechst. Scale bars: 10 μm. (E) The interaction between M and NSUN2 was predicted using Helixfold3 and visualized in pyMOL. The model depicts the M protein and NSUN2, with the N-terminal (amino acids 1–431) and C-terminal (amino acids 432–767) regions of NSUN2 distinguished for clarity. The rod-like structures represent hydrogen bonds and van der Waals forces within 3 Å. (F) Schematic diagram of truncated NSUN2. (G) HEK293T cells were co-transfected with pFlag-M, and either pMyc-NSUN2-N or pMyc-NSUN2-C and co-IP were performed using the indicated antibodies. (H) Western blot analysis of NSUN2 and M protein in NiV-infected Vero cells. (I) Immunohistochemistry detection of NSUN2 and M in lung tissues of NiV-infected hamster. Scale bars, 50 μm. (J) Density of NSUN2 positive cells in hamster lung tissues analyzed by Aipathwell. (K) HEK293T cells were transfected with varying amounts of pFlag-M (0, 2, 4, and 6 μg), with missing plasmids replenished using vector control. NSUN2 and Flag-M expression were analyzed by Western blot. (L) HEK293T cells transfected with vector or increasing amounts (0, 2, 4, and 6 µg) of pFlag-M were analyzed for NSUN2 mRNA levels by qRT-PCR. Data are presented as mean ± SEM (*n *= 3). ns: not significant, unpaired Student’s *t* test. (M) Ribosome-bound and input RNA from HEK293T cells transfected with vector or pFlag-M were analyzed for NSUN2 levels by qRT-PCR. Data are presented as mean ± SEM (*n *= 3). ns: not significant, unpaired Student’s *t* test. (N and O) HEK293T cells transfected with vector or pFlag-M were treated with 100 μg/mL CHX (N), 20 μmol/L MG132, 10 μmol/L CQ, or 5 mmol/L 3-MA (O) for 6 h. NSUN2 and Flag-M expression were analyzed by Western blot. IgG, immunoglobulin G; IP, immunoprecipitation; MS, mass spectrometry; NiV, Nipah virus; qRT-PCR, quantitative reverse transcription polymerase chain reaction; SEM, standard error of the mean.

The M protein plays a pivotal role in NiV replication and budding. To identify host factors that interact with M, we overexpressed Flag-tagged M in HEK293T cells and conducted immunoprecipitation (IP), followed by mass spectrometry (MS) analysis. This approach helped identify multiple host proteins, among which NSUN2 (ranked 11th) and YBX1 (ranked 32nd) exhibited robust interactions with M (Fig. 1A), implying a potential regulatory role of the m^5^C modification system in M protein function. To validate the interaction between NSUN2 and M protein, pNSUN2 was transfected into Vero cells before NiV infection. IP and MS analyses identified the M protein as the only viral protein interacting with NSUN2 (Figs. S1A and 1B), which was further confirmed via Co-IP and Western blot (Fig. 1C). Overexpression of the M protein induced the nuclear-to-cytoplasmic translocation of NSUN2, resulting in pronounced cytoplasmic co-localization of both proteins (Fig. 1D). These findings suggest a functional interplay between NSUN2 and the M protein.

Helixfold3 was employed to delineate the exact region of NSUN2 responsible for this interaction. The noncatalytic region of NSUN2 exhibited the highest binding affinity for the M protein (Fig. 1E). To further validate this, we constructed two truncated mutant plasmids: pMyc-NSUN2-N, containing the N-terminal methyltransferase catalytic domain and pMyc-NSUN2-C, encompassing the C-terminal region predicted to interact (Fig. 1F). Co-IP assays confirmed that the C-terminal region exhibited a stronger interaction with the M protein (Fig. 1G), consistent with the Helixfold3 predictions (Fig. S1B and S1C).

Given the interaction between NSUN2 and NiV M protein, we wondered whether viral infection modulates NSUN2 expression. NSUN2 levels were significantly upregulated in NiV-infected Vero cells, whereas the levels of other methyltransferases, such as DNMT2, ALYREF, and YBX1, remained unchanged (Fig. 1H). A similar increase in NSUN2 expression levels was observed in the lung tissues of NiV-infected hamsters (Fig. 1I and 1J). To determine whether this upregulation is mediated by M, a gradient of pFlag-M was transfected into Vero cells. NSUN2 protein levels increased in a dose-dependent manner along with M protein expression (Fig. 1K). However, neither NSUN2 mRNA abundance (Fig. 1L) nor its translation efficiency (Fig. 1M) was affected. Additionally, treatment with the ribosome inhibitor cycloheximide (CHX) did not diminish M protein–induced upregulation of NSUN2 (Fig. 1N), implying that the M protein enhances NSUN2 stability rather than promoting its protein synthesis. NSUN2 expression was analyzed by inhibiting the proteasomal, autophagic, or lysosomal degradation pathways employing MG132, chloroquine (CQ) or 3-methyladenine (3-MA), respectively. Only MG132 treatment prevented M-induced upregulation of NSUN2 (Fig. 1O). The findings indicate that the M protein enhances NSUN2 expression primarily by inhibiting proteasomal degradation.

### NSUN2 enhances NiV replication by promoting M expression and trafficking

**Figure 2.**
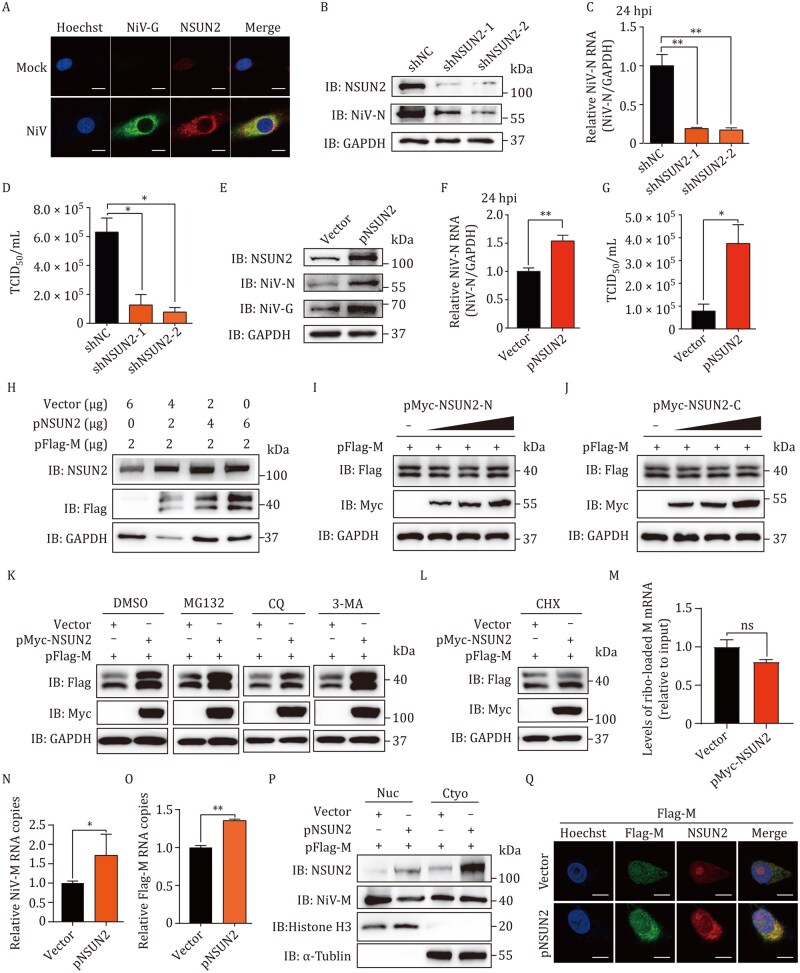
**NSUN2 enhances NiV replication by promoting M expression and trafficking**. (A) Confocal microscopy images of mock- or NiV-infected Vero cells stained with anti-NiV-G and anti-NSUN2 antibodies. Hoechst staining labels the nucleus. Scale bars: 10 μm. (B and E) Vero cells were treated with shRNA (B) or pFlag-NSUN2 (E), followed by NiV infection. NSUN2 and viral protein expression levels were analyzed by Western blot. (C and F) NiV RNA levels were measured by qRT-PCR in NSUN2 knockdown (C) or NSUN2-overexpression (F) Vero cells at 24 hpi. Data are presented as mean ± SEM (*n *= 3). *P ≤ 0.05, **P ≤ 0.01, unpaired Student’s *t* test. (D and G) Viral titers were determined by TCID_50_ assay in supernatants collected from Vero cells with NSUN2 knockdown (D) or NSUN2-overexpression (G) after NiV infection at 48 hpi. Data are presented by mean ± SEM (*n *= 3). *P ≤ 0.05, unpaired Student’s *t* test. (H–J) pFlag-M was co-transfected with varying amounts (0, 2, 4, and 6 μg) of pNSUN2 (H), pMyc-NSUN2-N (I) or pMyc-NSUN2-C (J). Missing plasmids were replaced with vectors. NSUN2 and Flag-M expression were analyzed by Western blot. (K and L) HEK293T cells transfected with vector or pMyc-NSUN2 were treated with 20 μmol/L MG132, 10 μmol/L CQ, 5 mmol/L 3-MA (K) or 100 μg/mL CHX (L) for 6 h. NSUN2 and Flag-M expression were analyzed by Western blot. (M) HEK293T cells transfected with vector or pMyc-NSUN2 were used to analyze ribosome-bound RNA for Flag-M. Data are mean ± SEM (*n *= 3). ns: not significant, unpaired Student’s *t* test. (N and O) Vector and pNSUN2 were transfected into Vero cells and infected with NiV (N) or transfected with pFlag-M (O). M RNA levels were quantified using qRT-PCR. (P) Nucleus and cytoplasm localization of M in vector- or pNSUN2-transfected Vero cells. Western blot analysis was performed on nuclear and cytoplasmic fractions using antibodies against M and NSUN2. Histone 3 and α-Tublin served as controls for each fraction. (Q) Vero cells co-transfected with pFlag-M and vector or pCDNA3.0-NSUN2 were treated with DMSO or MG132. Confocal microscopy images were obtained using anti-Flag and anti-NSUN2 antibodies. Hoechst staining marks the nucleus. Scale bars: 10 μm. NiV, Nipah virus; qRT-PCR, quantitative reverse transcription polymerase chain reaction; SEM, standard error of the mean.

Similar to most RNA viruses, NiV replicates in the cytoplasm, while NSUN2 is predominantly localized in the nucleus (Chellamuthu and Gray, 2020). To determine how the spatial barrier between NSUN2 and NiV replication was overcome, we examined the subcellular localization of NSUN2 following viral infection. Immunofluorescence analysis revealed that NSUN2 translocated from the nucleus to the cytoplasm after NiV-MY infection, where it co-localized with the viral G protein (Fig. 2A). In contrast, the translocation of DNMT2 and ALYREF was less pronounced compared with that of NSUN2 (Fig. S2A and S2B). Next, the effect of NSUN2 on NiV replication was investigated. NSUN2 knockdown in Vero cells or knockout in Huh7 cells reduced viral protein levels (Figs. 2B and S2C), lowered viral RNA abundance (Figs. 2C, S2D and S2E), and diminished progeny virus production (Figs. 2D and S2E) upon NiV-MY infection. Conversely, NSUN2 overexpression produced opposite effects (Figs. 2E–2G and S2F–H). A similar reduction in viral RNA was also observed in NSUN2-deficient cells infected with NiV-BD (Fig. S2I). In addition, a systematic assessment of the NSUN family showed that other m^5^C methyltransferases—including NSUN1 and NSUN3–7—did not affect NiV protein production, intracellular viral RNA levels, or viral RNA release (Fig. S3A–E), confirming that NSUN2 is the primary m^5^C methyltransferase governing NiV replication.

To determine whether NSUN2 modulated NiV replication by influencing M protein expression or its function, we assessed its effects in HEK293T cells. Overexpression of full-length NSUN2 significantly increased M protein levels (Fig. 2H). However, neither the M-interacting C-terminal domain nor the N-terminal catalytic domain of NSUN2 alone was sufficient to enhance M expression (Fig. 2I and 2J), suggesting that their direct interaction is not the sole determinant of this effect. Additionally, NSUN2 promoted M expression even in the presence of proteasomal (MG132), lysosomal (CQ), or autophagy (3-MA) inhibitors (Fig. 2K). Treatment with the ribosome inhibitor CHX abolished this effect (Fig. 2L), indicating that NSUN2 promotes the synthesis of M protein rather than enhancing its stability. Moreover, NSUN2 did not affect M RNA translation efficiency (Fig. 2M) but increased M RNA levels during NiV infection and pFlag-M transfection (Fig. 2N and 2O), indicating that NSUN2 transcriptionally regulated M protein expression.

Nuclear-cytoplasmic shuttling of the M protein is crucial for NiV replication (Pentecost et al., 2015; Wang et al., 2010). To investigate whether NSUN2 regulates M protein trafficking, we conducted nuclear-cytoplasmic fractionation and immunofluorescence assays. NSUN2 overexpression increased the cytoplasmic accumulation of the M protein (Fig. 2P and 2Q). Collectively, NSUN2 facilitates NiV replication potentially through dual mechanisms: enhancing M protein RNA transcription and regulating its nucleocytoplasmic transport.

### NSUN2 catalyzes m^5^C on NiV RNAs to promote M expression and viral replication

**Figure 3.**
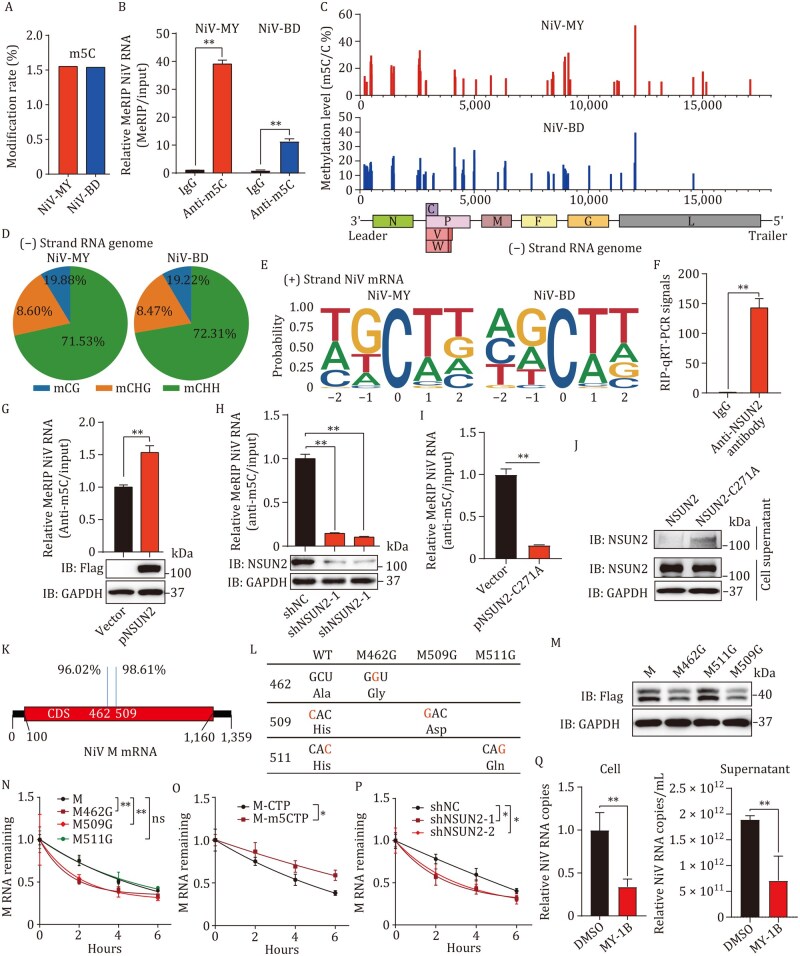
**NSUN2 mediates m^5^C modification of NiV RNA and enhances viral RNA stability**. (A) UHPLC-MS/MS analysis of NiV genome. Supernatants of NiV-MY and NiV-BD infected Vero cells were concentrated and enriched using dynabeads. The viral genome was extracted and analyzed by UHPLC-MS/MS. The y-axis represents the percentage of m^5^C among cytosine (C) residues. (B) MeRIP-qRT-PCR analysis. RNA extracted from NiV-infected Vero cells was incubated with IgG or anti-m^5^C antibodies and subjected to IP and qRT-PCR. Data are presented as mean ± SEM (*n *= 3). **P ≤ 0.01, unpaired Student’s *t* test. (C) bsRNA-Seq analysis. NiV-MY and NiV BD genomic RNAs from viral-infected Vero cell supernatants were analyzed by bsRNA-Seq. The vertical axis represents the probability of m^5^C methylation at each C site, with sites showing an average probability of 10 or higher displayed. Data are derived from *n *= 3 independent experiments. (D and E) Bioinformatics analysis. Sequence distribution and conserved sequence of m^5^C in the NiV-MY and NiV-BD mRNA. (F) Binding of NSUN2 to NiV RNA. NiV-infected cells were crosslinked by formaldehyde, followed by IP using anti-NSUN2 antibodies and quantified by qRT-PCR. IgG served as a negative control. Data are presented as mean ± SEM (*n *= 3). **P ≤ 0.01, unpaired Student’s *t* test. (G − I) MeRIP-qRT-PCR analysis of m^5^C level in NiV RNA from cells treated with pNSUN2 (G), shRNA (H), or pNSUN2-C271A (I). Data are represented as mean ± SEM (*n *= 3). **P ≤ 0.01, unpaired Student’s *t* test. (J) Cells transfected with pNSUN2 or pNSUN2-C271A were infected with Nipah virus (NiV). NSUN2 expression was analyzed by Western blot in both cell lysates and purified viral particles from the supernatant. (K and L) Schematic representation of M and m^5^C mutants. (M) The expression of M and m^5^C mutants in transfected HEK293T cells was detected by Western blot. (N and O) The mRNAs of M and m^5^C mutants were transcribed by T7 polymerase with CTP or m^5^CTP as a substrate and transfected into HEK293T cells. RNA levels were quantified by qRT-PCR at the indicated time points (0 h represents 4 h after transfection). (P) M mRNA was transfected into HEK293T cells treated with NSUN2 shRNA, and M RNA levels were detected by qRT-PCR. (Q) Vero cells were treated with 10 μmol/L MY-1B, followed by NiV infection. Viral RNA levels in both cells and the supernatant at 48 hpi were quantified using qRT-PCR. Data are presented as mean ± SEM (*n *= 3). **P ≤ 0.01, unpaired Student’s *t* test. IP, immunoprecipitation; MeRIP-qRT-PCR, methylated RNA immunoprecipitation-quantitative reverse transcription polymerase chain reaction; NiV, Nipah virus; qRT-PCR, quantitative reverse transcription polymerase chain reaction; UHPLC-MS/MS, ultra-high performance liquid chromatography-tandem mass spectrometry.

NSUN2-mediated m^5^C modification is critical for regulating RNA expression and protein translation in cellular and viral RNAs (Tsai and Cullen, 2020). To investigate the m^5^C modification in NiV RNAs, we conducted a UHPLC-MS/MS (ultra-high performance liquid chromatography-tandem mass spectrometry) analysis. Genomic RNAs from both NiV-MY and NiV-BD virions contained approximately 1.5% m^5^C modifications (Fig. 3A), which is significantly higher than that observed in host mRNAs (0.03%–0.1%) (Huber et al., 2015; Legrand et al., 2017; Wiener and Schwartz, 2020). Additionally, NiV RNAs were enriched using anti-m^5^C antibody, further confirming the presence of m^5^C modifications (Fig. 3B). To comprehensively map m^5^C modifications in NiV negative-strand genomic RNAs, bisulfite RNA sequencing (bsRNA-seq) was conducted on genomic RNAs extracted from virus particles in the supernatants of NiV-MY- and NiV-BD-infected cells. Bioinformatics analysis revealed that m^5^C modifications were distributed throughout the genomes of both strains, with the 3′ end of the NiV-BD genome exhibiting lower modification levels than that of NiV-MY (Fig. 3C). The m^5^C motifs, including CG, CHG, and CHH contexts, were similar between the strains (Fig. 3D), with the CAA sequence being the most prevalent (Fig. S4A). To further validate m^5^C modifications in NiV positive-strand mRNAs, we employed nanopore direct RNA sequencing (DRS), which allows for the direct analysis of polyadenylated RNA, thereby eliminating interference from the negative-strand genomic RNA. Widespread m^5^C modifications were observed in the mRNAs of both NiV-MY and NiV-BD (Fig. S4B), with conserved m^5^C motifs exhibiting only minor differences in the nucleotide adjacent to the methylated cytosine (Fig. 3E).

NiV does not encode a methyltransferase, which indicates that m^5^C modifications on viral RNAs are likely mediated by NSUN2. RNA IP (RIP) assays confirmed the interaction between NSUN2 and NiV RNAs (Fig. 3F). Overexpression of NSUN2 significantly enhanced m^5^C modification levels on NiV RNA (Fig. 3G), while NSUN2 knockdown reduced these modifications (Fig. 3H). Moreover, transfection with NSUN2-C271A, a mutant that prevents NSUN2 dissociation from RNA and thus impedes sustained m^5^C formation (Courtney et al., 2019a), resulted in reduced m^5^C modifications on NiV RNAs (Fig. 3I), and NSUN2-C271A was also incorporated into viral particles (Fig. 3J). Overexpression of the catalytic mutant NSUN2-C321A did not enhance m5C modification on NiV RNAs (Fig. S4C). These findings indicate that NSUN2 catalyzes m^5^C modifications on NiV RNAs.

The M RNA contains two m^5^C sites at nucleotide positions 462 and 509 (Fig. 3K). To determine whether NSUN2 enhances M RNA levels through m^5^C modifications, three mutant plasmids were generated by substituting cytosine (C) into guanine (G) at these positions: pFlagM-C462G and pFlagM-C509G (Fig. 3L). The pFlagM-C511G mutant was constructed by mutating a neighboring nonmethylated cytosine (nt511) to G as a negative control (Fig. 3L). Transfecting these mutants into cells followed by WB analysis revealed that mutations at the m^5^C sites reduced M protein expression compared with the negative control (Fig. 3M). To further investigate the influence of m^5^C modifications on M expression, M RNA and its mutant variants were synthesized via T7 *in vitro* transcription using CTP or m^5^CTP as substrates and then transfected into cells. M RNAs with mutations at the m^5^C sites degraded more rapidly (Fig. 3N), while those transcribed with m^5^CTP exhibited increased stability (Fig. 3O). Furthermore, NSUN2 knockdown resulted in reduced M RNA stability (Fig. 3P). Ribosome binding assays showed that mutation of the m^5^C site had no effect on translation efficiency (Fig. S4D). Collectively, NSUN2 stabilizes M RNAs through m^5^C modifications, leading to increased M RNA abundance and protein expression.

To confirm that NSUN2’s impact on NiV replication depends on its m^5^C writing activity, Vero cells were treated with MY-1B, an m^5^C methylation inhibitor that selectively binds to the cysteine residue (C271) in the active site of NSUN2 (Tao et al., 2023) and then infected with NiV. MY‑1B treatment resulted in a reduction of viral RNA copies in both infected cells and culture supernatants under conditions where MY-1B exhibited no detectable cytotoxicity (Figs. 3Q and S4E), indicating that the m5C catalytic function of NSUN2 regulates NiV replication.

### NSUN2 facilitates M nuclear export via enhanced ubiquitination

**Figure 4.**
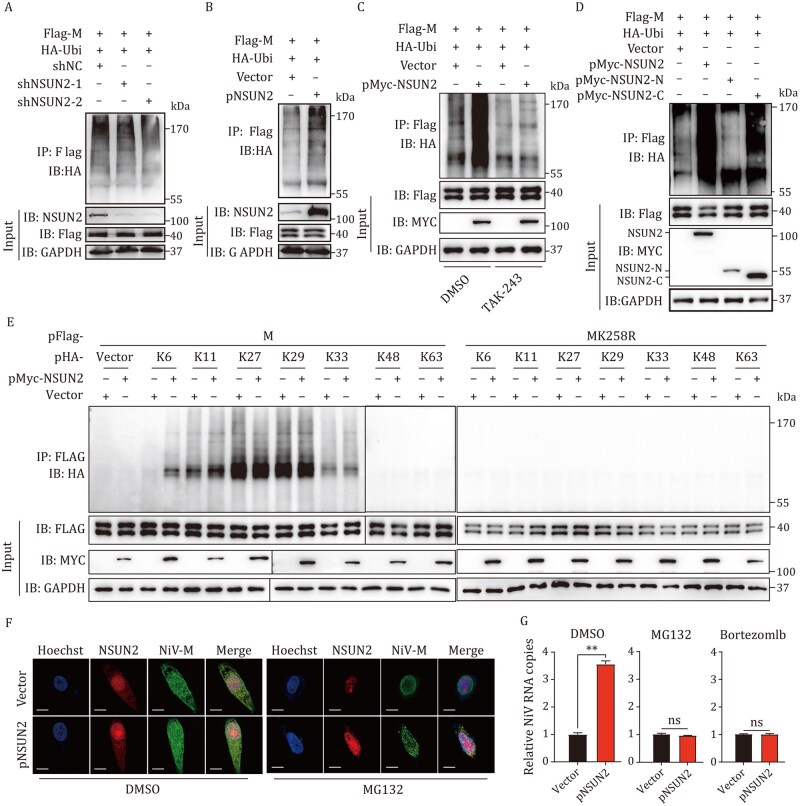
**NSUN2 promotes M nuclear export through ubiquitination modification**. (A and B) Ubiquitination assay. HEK293T cells were co-transfected with pFlag-M and pHA-Ubi following NSUN2 knockdown (A) or overexpression (B). IP and Western blot analysis were performed using the indicated antibodies. (C) HEK293T cells were co-transfected with pFlag-M and either vector or pNSUN2, followed by treatment with DMSO or TAK-243 (500 nmol/L) for 6 h. M ubiquitination was detected by IP and Western blot. (D) HEK293T cells were co-transfected with pFlag-M, pHA-Ubi and either pNSUN2 or its mutants. M ubiquitination was detected by IP and Western blot. (E) HEK293T cells were co-transfected with pFlag-M or pFlag-MK258R, along with either vector or pNSUN2, pHA-K6, -K11, -K27, -K29, -K33, -K48 or -K63. IP and immunoblot analysis were performed using the indicated antibodies. (F) Vero cells co-transfected by pFlag-M and either vector or pCDNA3.0-NSUN2, then treated with DMSO or MG132. Confocal microscopy was performed using anti-Flag antibodies and anti-NSUN2, with Hoechst staining for the nucleus. Scale bars: 10 μm. (G) qRT-PCR was used to quantify NiV RNA levels in Vero cells transfected with either vector or pNSUN2 and treated with DMSO, MG132 or bortezomib. Data are presented as mean ± SEM (*n *= 3). **P ≤ 0.01, ns: not significant, unpaired Student’s *t* test. IP, immunoprecipitation; NiV, Nipah virus; qRT-PCR, quantitative reverse transcription polymerase chain reaction.

Ubiquitination plays a key role in the nuclear-cytoplasmic trafficking of M proteins and facilitates viral budding (Pentecost et al., 2015; Wang et al., 2010). Given that NSUN2 enhances the nucleocytoplasmic shuttling of M (Fig. 2P and 2Q), its potential influence on M ubiquitination was examined. Ubiquitination assays demonstrated that NSUN2 silencing reduced M ubiquitination (Fig. 4A), while NSUN2 overexpression had the opposite effect (Fig. 4B). Treatment with the ubiquitination inhibitor TAK-243 decreased M ubiquitination and abolished the enhancement induced by NSUN2, indicating that NSUN2 facilitates M protein ubiquitination (Fig. 4C). To identify the key regulatory domain of NSUN2 responsible for M ubiquitination, truncated mutant plasmids, pMyc-NSUN2-N and pMyc-NSUN2-C, were transfected into cells, followed by ubiquitination assays. pNSUN2-C transfection resulted in increased M ubiquitination, with no detectable effect observed when pNSUN2-N was transfected (Fig. 4D). These findings imply that NSUN2 promotes M ubiquitination through a direct interaction with M. Ubiquitin chain linkage types include K6, K11, K27, K29, K33, K48, and K63. To determine which linkages are regulated by NSUN2, HEK293T cells were co-transfected with pFlag-M and plasmids encoding HA-tagged ubiquitin mutants specific to each linkage type. The ubiquitination-deficient mutant Flag-MK258R served as a negative control. NSUN2 specifically enhanced K6- and K11-linked ubiquitination of the M protein, with no observable effects on other linkages (Fig. 4E).

To further explore how NSUN2 regulates M protein nuclear export through ubiquitination, NSUN2-overexpressing cells were treated with either DMSO or the proteasome inhibitor MG132. NSUN2 promoted M protein nuclear export in DMSO-treated cells, but this effect was abolished in MG132-treated cells (Fig. 4F). Thus, NSUN2-induced cytoplasmic trafficking of M protein is dependent on its ubiquitination. Ubiquitination of M protein is closely associated with the NiV replication (Pentecost et al., 2015; Wang et al., 2010). NSUN2 may regulate NiV replication by modulating M protein ubiquitination. Therefore, the effects of proteasome inhibitors on NiV infection were evaluated. MG132 and bortezomib treatment impaired the NSUN2-mediated enhancement of viral replication compared with that of DMSO control (Fig. 4G). Collectively, NSUN2 facilitates NiV replication through a ubiquitination-dependent mechanism, which could be effectively disrupted by proteasome inhibitors.

### NSUN2 boosts M ubiquitination by promoting GNB2-M interaction

**Figure 5.**
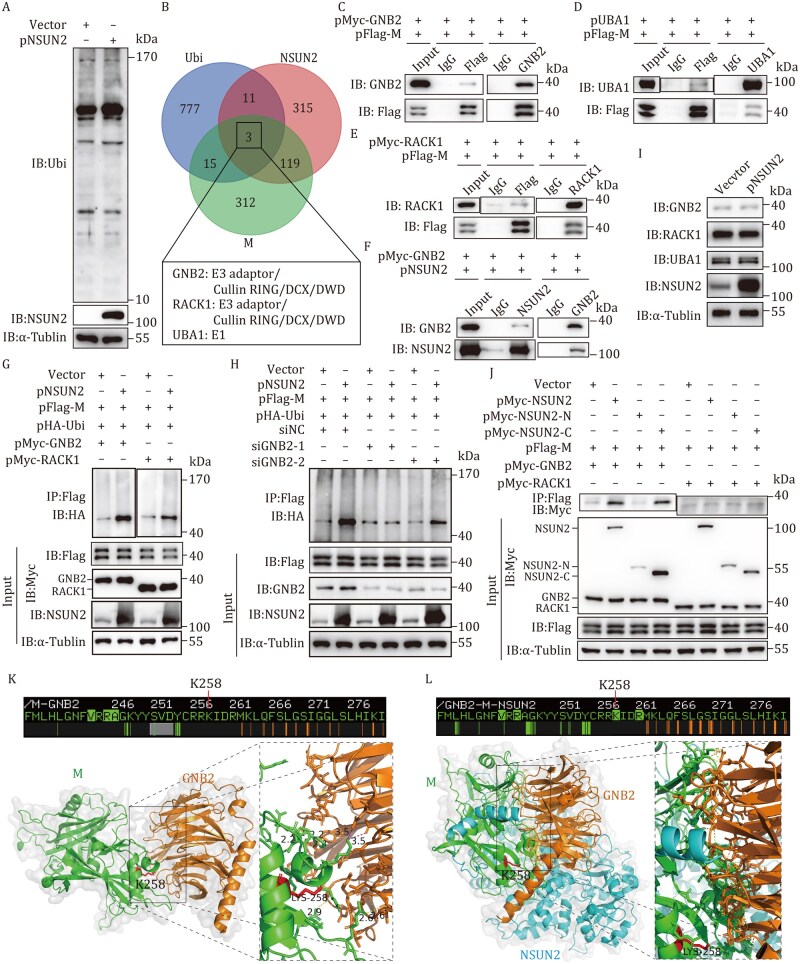
**NSUN2 enhances M ubiquitination by promoting GNB2 and M interaction**. (A) ubiquitination levels of proteins in HEK293T cells transfected with vector or pNSUN2. (B) Venn diagram illustrating the overlap among NSUN2-interacting proteins (from NSUN2 IP-MS), M-interacting proteins (from Flag-M IP-MS), and ubiquitination-related proteins from the ubiquitin-proteome database. (C–F) HEK293T cells were co-transfected with various combinations of pFlag-M, pNSUN2, pGNB2, pRACK1, or pUBA1, followed by IP and Western blot to validate interactions among M, NSUN2, GNB2, RACK1, and UBA1. (G and H) Ubiquitination assays demonstrating that NSUN2 promotes M ubiquitination in the presence of GNB2 and RACK1 (G) while knocking down GNB2 (H) reduces NSUN2-mediated M ubiquitination. (I) HEK293T cells were transfected with pNSUN2 to detect the expression of RACK1, UBA1 and GNB2. (J) Co-IP analysis showing the binding of M to GNB2 or RACK1 in HEK293T cells under NSUN2, NSUN2-N, or NSUN2-C overexpression, confirming the role of NSUN2 in facilitating M-GNB2 interaction. (K and L) Prediction and visualization of the M-GNB2 interaction with or without NSUN2 using Helixfold3. The top panel delineates amino acids proximal to K258 on the M protein, with shaded residues indicating interfacial residues involved in GNB2 interactions. Vertical lines beneath the residues denote all interfacial residues across both interacting proteins. In the structural schematic below, K258 of the M protein is highlighted in red using stick representation, while interfacial residues are explicitly rendered as sticks. The rod-like structures represent hydrogen bonds and van der Waals forces within 3 Å. IP, immunoprecipitation; MS, mass spectrometry.

As NSUN2 lacks intrinsic ubiquitination activity, we speculated that it may indirectly regulate M protein ubiquitination by enhancing the activity of ubiquitin-related proteins or facilitating their interaction with M protein. To test this, we first examined global ubiquitination levels under NSUN2 overexpression, which showed no significant change in the overall ubiquitination levels (Fig. 5A), suggesting that NSUN2 does not broadly affect the ubiquitination of catalytic machinery. Thus, we hypothesized that NSUN2 modulates ubiquitination by promoting the binding of specific ubiquitin-related proteins to M protein. Mass spectrometry analysis of NSUN2- and M-interacting proteins, aligned with ubiquitination database entries (Zhou et al., 2018), identified three candidate proteins: GNB2, RACK1, and UBA1 (Fig. 5B). Co-IP assays confirmed the interactions between each of these proteins and either NSUN2 or M protein (Figs. 5C–F and S5A).

To determine which proteins are involved in NSUN2-mediated M protein ubiquitination, we overexpressed or silenced GNB2, RACK1, or UBA1 individually. Overexpression of any of these proteins did not impair the ability of NSUN2 to enhance M ubiquitination (Figs. 5G and S5B). However, silencing any of these candidates reduced NSUN2-mediated increase in M protein ubiquitination (Figs. 5H, S5C and S5D), indicating that all three proteins contribute to this process. Next, we examined whether NSUN2 influences the expression levels of GNB2, RACK1, and UBA1. NSUN2 overexpression did not alter the expression of these proteins (Fig. 5I), demonstrating that NSUN2 probably modulates their interaction with M protein rather than their abundance. Consistent with this notion, co-IP experiments revealed that overexpression of NSUN2 or pNSUN2-C enhanced the interaction between the M protein and GNB2 (Fig. 5J), while the correlation between the M protein and either RACK1 or UBA1 remained unchanged (Figs. 5J and S5E). Helixfold3 predictions indicated that, in the absence of NSUN2, the number of amino acids mediating the interaction between the M protein and GNB2 was limited (Fig. 5K). However, in the presence of NSUN2, the number of interacting amino acids increased significantly, particularly those surrounding K258—the ubiquitination site on the M protein (Fig. 5L). However, NSUN2 did not enhance the interaction between M and RACK1 or and UBA1 (Fig. S5F–I). Collectively, the results demonstrate NSUN2 promotes M protein ubiquitination by facilitating the binding of GNB2 to M.

### GNB2 promotes M ubiquitination by recruiting the E3 ligase TRIM28

**Figure 6.**
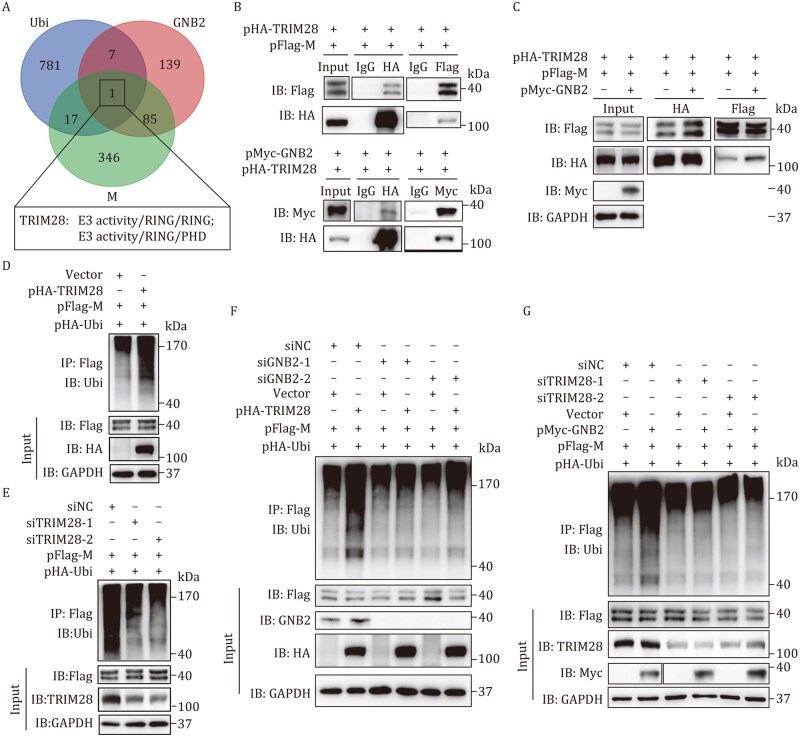
**GNB2 promotes M ubiquitination by recruiting the E3 ligase TRIM28**. (A) Myc-tagged GNB2 was immunoprecipitated under 300 mmol/L salt conditions and analyzed by mass spectrometry to identify ubiquitination-related interacting proteins, and a Venn diagram highlights those proteins that also interact with M. (B) Co-IP assays validating the interactions among GNB2, TRIM28, and M in HEK293T cells. (C) Co-IP assays showing increased association between TRIM28 and M in the presence of GNB2 overexpression. (D and E) Ubiquitination assays analyzing the effect of TRIM28 overexpression (D) or TRIM28 knockdown (E) on M ubiquitination in HEK293T cells. (F) Ubiquitination analysis showing the effect of GNB2 knockdown on TRIM28-induced M ubiquitination. (G) Ubiquitination analysis showing the effect of TRIM28 knockdown on GNB2-induced M ubiquitination. IP, immunoprecipitation.

GNB2 acts as an adaptor without intrinsic ubiquitin ligase activity, suggesting that additional E3 ligases are required to facilitate M ubiquitination. To identify such factors, Myc-tagged GNB2 was immunoprecipitated and analyzed by MS. Among the ubiquitination-related proteins enriched in the GNB2 interactome, TRIM28 was the only one also present in the previously defined M interactome (Fig. 6A), highlighting it as a candidate linking GNB2 and M. Co-IP confirmed that TRIM28 interacted with both GNB2 and M (Fig. 6B), and GNB2 overexpression strengthened the TRIM28–M interaction (Fig. 6C), indicating that GNB2 facilitates the recruitment of TRIM28 to the M protein.

Whether TRIM28 was involved in M ubiquitination was next investigated. Overexpression of TRIM28 enhanced M ubiquitination (Fig. 6D), whereas knockdown TRIM28 led to the opposite effect (Fig. 6E), implying that TRIM28 acts as a positive regulator of M ubiquitination. The role of GNB2 in TRIM28-mediated M ubiquitination was further examined. Notably, depletion of GNB2 impaired the TRIM28-mediated enhancement of M ubiquitination (Fig. 6F). Loss of TRIM28 attenuated the effect of GNB2 overexpression (Fig. 6G). Overall, the above results indicate that both GNB2 and TRIM28 are required for efficient M ubiquitination. GNB2 facilitates M ubiquitination by promoting TRIM28 engagement with M.

### Proteasome and m^5^C inhibitors as a dual‑target antiviral strategy against NiV

**Figure 7.**
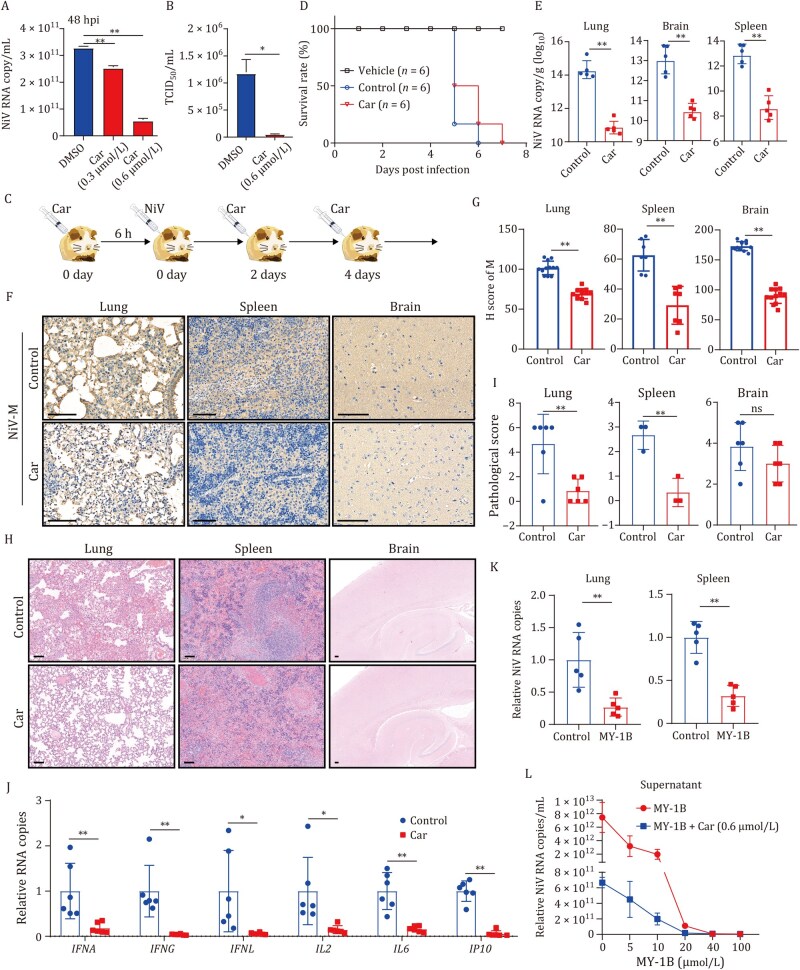
**Proteasome and methylation inhibitors suppress NiV replication**. (A and B) Vero cells pretreated with DMSO or Car were infected with NiV. Viral RNA copy numbers (A) and progeny virus titers (B) in the supernatants were quantified using qRT-PCR and TCID_50_ assays, respectively. (C) Experimental design for NiV infection and drug administration in hamsters. Hamsters were intraperitoneally injected with NiV-MY (200 LD_50_) and treated with or without Car. (D) Survival curves of hamsters treated with or without Car following intraperitoneal challenge with a lethal NiV-MY dose (500 LD_50_). (E) Viral RNA copy numbers in the lung, brain, and spleen tissues of Car-treated hamsters were quantified using qRT-PCR at 4 days postinfection (dpi). (F) Immunohistochemistry analysis of NiV M protein in the brain, lung, and spleen of NiV-MY-infected hamsters at 96 hpi. Scale bars: 100 μm. (G) Quantitative analysis of positive cell density in hamster tissues using Aipathwell software. (H) Hematoxylin and eosin (H&E) staining of hamster tissues, showing histopathological changes in the lung and spleen. Scale bars: 100 μm. (I) Pathological grading of H&E-stained tissues using a four-level scoring system to assess lesion severity. (J) Cytokine expression in lung tissues of control or Car-treated hamsters 4 days after NiV infection was measured via qRT-PCR. (K) qRT‑PCR quantification of viral RNA copies in lung, brain, and spleen tissues of hamsters treated with MY‑1B at 4 days post‑infection. (L) Vero cells were treated with different concentrations of MY-1B, either alone or in combination with Car, followed by NiV infection. Viral RNA levels in the culture supernatant at 48 hpi were quantified using qRT-PCR. NiV, Nipah virus; qRT-PCR, quantitative reverse transcription polymerase chain reaction.

Currently, no effective antiviral drugs are available for NiV. Given that NSUN2-mediated M protein ubiquitination enhances viral replication, targeting this pathway could offer a potential therapeutic strategy. Proteasome inhibitors, such as MG132 and bortezomib, disrupt the proviral activity of NSUN2 (Fig. 4G) and suppress viral replication (Fig. S6A and S6B). However, owing to the significant side effects associated with bortezomib (Moreau et al., 2011), we evaluated carfilzomib (Car), a next-generation proteasome inhibitor approved for clinical application. Treatment with as low as 0.6 μmol/L Car reduced NiV RNA levels by 5-fold and viral titers by 10-fold, as quantified via quantitative reverse transcription polymerase chain reaction (qRT-PCR) and TCID_50_ (50% tissue culture infectious dose) assays (Figs. 7A, 7B, S6C and S6D).

The antiviral effects of Car were further evaluated in a hamster infection model using the NiV-MY strain. Six-month-old hamsters received intraperitoneal injections of Car at various concentrations every other day (Fig. 7C). A dose of 4 mg/kg Car was selected for infection experiments, as it represented the highest concentration tested without significantly affecting body weight (Fig. S6E). Compared with control animals, Car-treated hamsters exhibited prolonged survival (Fig. 7D) and significantly lower viral RNA copy numbers in the spleen, lungs, and brain (Fig. 7E). Immunohistochemistry (IHC) analysis demonstrated reduced viral replication in the spleen, lung, and brain of Car-treated hamsters (Fig. 7F and 7G; Table S1). Hematoxylin and eosin (HE) staining revealed that Car-treated hamsters exhibited marked attenuation of multiorgan pathological manifestations compared with untreated controls, which showed characteristic splenic lesions (focal necrosis, lymphocytic infiltration, hemorrhage, and loss of red/white pulp demarcation) and pulmonary abnormalities (alveolar wall thickening, hemorrhagic foci, and perivascular edema) (Fig. 7H and 7I; Tables S2 and S3). Although brain lesions were less pronounced, the Car-treated group exhibited milder symptoms compared with those of the controls (Fig. 7H and 7I; Table S4). As inflammatory responses play a key role in NiV pathogenesis, the expression of inflammatory cytokines was measured. Car treatment reduced mRNA levels of *IFNA*, *IFNG*, *IFNL*, *IL2*, *IL6*, and *IP10* in the lungs and brain (Figs. 7J and S6F). Collectively, these findings demonstrate that Car exerts potent anti-NiV effects *in vivo*.

To evaluate the *in vivo* antiviral efficacy of the m^5^C methylation inhibitor MY-1B against NiV infection, we followed the same experimental protocol as for Car, wherein hamsters received 15 mg/kg MY-1B prior to NiV-MY or NiV-BDchallenge (Fig. S6G). Compared with the control group, MY-1B administration reduced viral RNA copies in both lung and spleen tissues (Figs. 7K and S6H), indicating effective inhibition of NiV replication *in vivo*. To further test NSUN2’s antiviral role *in vivo*, tamoxifen-induced *Nsun2* conditional knockout (cKO) mice and Cre-negative littermates (Fig. S7A–E) were challenged intraperitoneally with NiV-MY (1000 LD_50_). At 4 dpi, *Nsun2*-cKO mice showed consistently lower viral RNA levels in brain, lungs, and spleen compared with controls (Fig. S7F). Moreover, co-administration of 0.6 μmol/L Car with escalating MY-1B concentrations produced dose-dependent enhancement of antiviral activity, and the superior therapeutic outcomes achieved through dual inhibitor therapy relative to MY-1B monotherapy (Fig. 7L). This reciprocal enhancement underscores a synergistic effect between methylation and proteasome inhibitors in suppressing NiV replication, highlighting NSUN2 as a promising therapeutic target for NiV and offering a potential dual-targeting antiviral strategy for the treatment of NiV infections.

## Discussion

**Figure 8.**
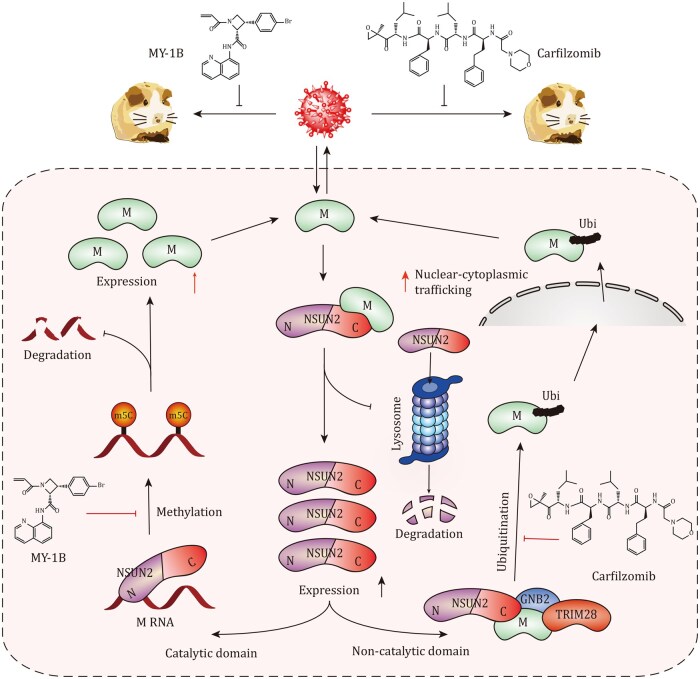
**Model of NSUN2-mediated dual regulation of Nipah virus replication**. Upon NiV infection, the viral M protein interacts with and stabilizes host NSUN2 by preventing its proteasomal degradation. NSUN2 enhances viral RNA stability through m^5^C methylation, facilitating increased expression of the M transcript. Concurrently, NSUN2 serves as a scaffold to recruit the E3 adaptor GNB2, which in turn enhances the association of the E3 ligase TRIM28 with the M protein, thereby promoting M ubiquitination and facilitating its nuclear export. These coordinated actions support viral replication and particle assembly. Pharmacological interference with either the RNA modification or ubiquitin-mediated pathways effectively attenuates NiV propagation *in vitro* and in animal models. NiV, Nipah virus.

This study reveals a previously unrecognized dual mechanism by which the host m^5^C methyltransferase NSUN2 is co-opted by NiV to enhance viral replication. Acting as both an epitranscriptomic writer and a scaffolding regulator, NSUN2 stabilizes viral transcripts via m^5^C modification and facilitates posttranslational processing of the matrix (M) protein by recruiting the E3 adaptor GNB2, which enhances TRIM28-mediated ubiquitination (Fig. 8). These two distinct but convergent functions collectively support efficient viral replication and virion assembly. More broadly, our findings underscore how a single host factor can be hijacked through multilayered strategies to coordinate both RNA-level and protein-level regulation, offering additional possibilities for therapeutic intervention.

The abundance and distribution of m^5^C modifications in NiV genomic RNAs and mRNAs differ from those in host and other viral RNAs. In host mRNAs, m^5^C accounted for 0.03% to 0.1% of total cytidine (Huber et al., 2015; Legrand et al., 2017; Wiener and Schwartz, 2020). In positive-strand RNA viruses such as murine leukemia virus, HIV-1, Zika virus, dengue virus, hepatitis C virus, and papillomavirus, m^5^C levels were approximately 0.54%, 0.0671%, 0.178%, 0.295%, 0.27%, and 0.181%, respectively (Courtney et al., 2019a; McIntyre et al., 2018). m^5^C modification levels in positive-strand viral RNAs are generally higher than those in host RNAs. Nevertheless, our study revealed that both the negative-strand RNA genome and positive-strand mRNA of NiV contain m^5^C modifications, with genomic content reaching approximately 1.55%, which is higher than those in host mRNAs and the genomes of several positive-strand RNA viruses. This elevated m^5^C modification in NiV suggests a significant effect on the virus and indicates that the m^5^C modification system could be a potential target for antiviral therapies. Our comparative profiling further revealed that NiV-MY and NiV-BD exhibit distinct m^5^C landscapes: NiV-MY shows enriched genomic m^5^C near the 3′ region, whereas NiV-BD contains higher m^5^C levels in corresponding viral mRNAs.

RNA modifications are closely linked to viral pathogenesis. Inhibition of NSUN2 markedly attenuated disease severity *in vivo*, indicating that NSUN2-dependent m^5^C methylation contributes to NiV pathogenicity beyond merely supporting viral replication. Consistent with this notion, accumulating evidence has implicated m^5^C modifications in shaping viral virulence across diverse pathogens. For example, NSUN2-mediated m^5^C enhances HBV RNA stability and exacerbates liver pathology in animal models (Feng et al., 2023). Recent studies of Japanese encephalitis virus (JEV) show that infection-driven stabilization of NSUN2 remodels both host and viral m^5^C patterns to suppress antiviral signaling and facilitate persistent infection (Chen et al., 2025). Notably, NiV-MY RNA exhibits higher m^5^C levels than BD, correlating with its enhanced replication in both cultured cells and hamsters (Luo et al., 2025). Pharmacological inhibition of m^5^C in MY-1B caused a more pronounced reduction in replication, indicating that m^5^C abundance directly impacts viral fitness. Together, these findings link strain-specific m^5^C deposition to differences in pathogenicity, emphasizing m^5^C as a key regulator of NiV virulence.

The methyltransferase NSUN2 regulates NiV replication through mechanisms that extend beyond its traditional role in RNA modification. While previous research has established that methyltransferases, such as METTL3 and NSUN2, are crucial for viral replication by modulating RNA modification (Ding et al., 2024; Hao et al., 2019), our findings reveal that NSUN2 also facilitates NiV replication through an RNA modification-independent pathway. Specifically, the C-terminal domain of NSUN2 interacts with the NiV M protein and enhances its ubiquitination, a process essential for M protein nuclear export and viral budding. This discovery broadens the functional repertoire of RNA-modifying enzymes, highlighting their ability to regulate viral replication through RNA and protein modifications. This dual functionality is not unique to NSUN2. For instance, METTL3 interacts with the RNA-dependent RNA polymerase (3D) of enterovirus 71 and host protein DDX3X, enhancing 3D ubiquitination while suppressing that of DDX3X (Hao et al., 2019; Hao et al., 2024). Similarly, NAT10, another RNA-modifying enzyme, exhibits dual roles in acetylating RNA and histones (Hao et al., 2022). These findings imply that RNA-modifying proteins may function as multifunctional regulators, influencing RNA metabolism and protein posttranslational modifications. This paradigm shift is further supported by findings showing that NSUN2 knockdown reduces replication in certain viruses without affecting their RNA m^5^C modification levels (Xiong et al., 2024; Zhang et al., 2022), indicating that the noncatalytic functions of these enzymes may be equally critical for viral replication.

Ubiquitination is important for NiV M protein trafficking and virus assembly. However, the precise mechanisms governing M protein ubiquitination, particularly the identity of the key E3 components and the specific ubiquitin chain linkages involved, remain poorly understood. We identified the NSUN2–GNB2–TRIM28 axis that promotes K6/K11-linked ubiquitination of the M protein, which implied that M ubiquitination can be shaped not only by canonical ubiquitin enzymes but also by RNA-modification–associated regulatory factors. Notably, this crosstalk between RNA epitranscriptomic regulation and ubiquitin signaling plays a critical role in henipavirus infection. Together, these findings provide a more integrated view of how M ubiquitination is orchestrated and underscore the need for future studies to define the full spectrum of enzymes, linkage types, and regulatory mechanisms involved.

The clinical relevance of our findings is highlighted by the observation that Car—a clinically approved proteasome inhibitor—reduces NiV replication and pathology in hamsters. Unlike direct-acting antivirals, which are prone to rapid resistance (Dawes et al., 2018), host-directed agents including Car target evolutionarily conserved host pathways. Car monotherapy achieved a 10-fold reduction in viral titers (Fig. 6B), surpassing the efficacy of previously tested NiV inhibitors including ribavirin (Georges-Courbot et al., 2006) and this likely results from its dual blockade of NSUN2 stabilization and M protein ubiquitination. The synergistic antiviral effect observed with the m^5^C methylation inhibitor MY-1B underscores the therapeutic potential of co-targeting NSUN2 enzymatic and scaffolding functions. Given that MY-1B selectively inhibits NSUN2 by covalently binding to C271 (Tao et al., 2023), combining Car with MY-1B may enhance antiviral potency while reducing off-target effects.

Collectively, our study identified NSUN2 as a key host factor exploited by NiV to promote viral replication through mechanisms involving mRNA stabilization, m^5^C modification, and ubiquitination. The interaction between NSUN2 and the M protein exemplifies the intricate interplay between host and pathogen, provides new insights into NiV pathogenesis, and reveals promising antiviral targets. Future research should explore the broader relevance of NSUN2 to paramyxoviruses, clarify the structural basis of the NSUN2–GNB2–TRIM28-M complex (e.g., via cryo-electron microscopy), and develop inhibitors targeting protein–protein interaction. Additionally, the therapeutic potential of the Car/MY-1B combination requires further validation in nonhuman primates, particularly considering the NiV neurotropism and the challenges posed by the blood–brain barrier on drug delivery.

## Supplementary Material

pwag003_Supplementary_Data

## Data Availability

The data supporting this study are included in the paper and Supplementary Information, and additional data are available from the corresponding author upon reasonable request.

## Artificial intelligence

We used AI-assisted technologies, including Helixfold3 (Baidu) for protein structure prediction and DeepSeek solely for language polishing of the manuscript. All scientific content, analyses, and conclusions were conceived, performed, and verified by the authors.
