# Supplementary material for: NSUN2‑mediated epitranscriptomic and ubiquitin modulation of Nipah virus matrix protein reveals a dual-targeting antiviral strategy
Source: Protein Cell. 2026 Feb 12;17(7):644–61. doi: 10.1093/procel/pwag003 (PMC13340930; doi:10.1093/procel/pwag003)
Supplement: pwag003_Supplementary_Data [file pwag003_supplementary_data.pdf]

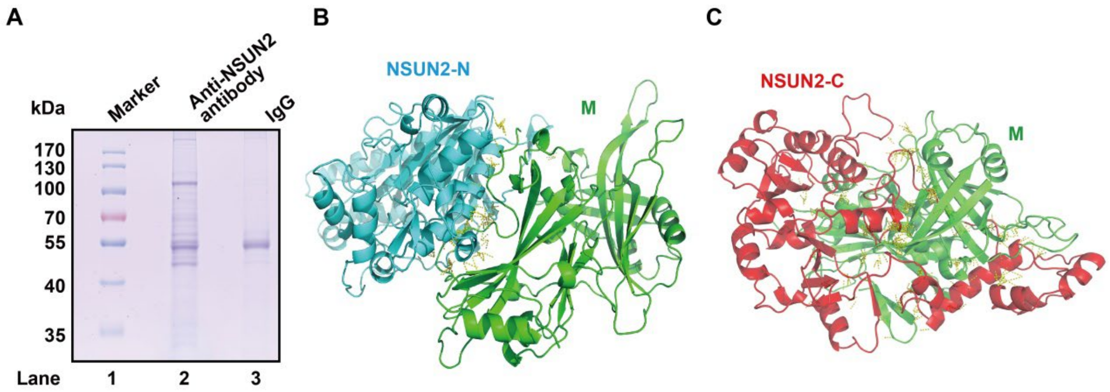

Figure S1. NSUN2 interacts with M protein. (A) NiV-infected Vero cells were immunoprecipitated with anti-NSUN2 or IgG antibodies, and then the proteins were separated by polyacrylamide gel electrophoresis-and stained with Coomassie Blue. The gel slices were excised for mass spectrometry analysis. (B & C) The potential interactions between the M protein and NSUN2-N (B) or NSUN2-C (C) domains of NSUN2 were predicted using Helixfold3 and visualized using PyMOL. In the graphical representation, the M protein is depicted as a green chain, the NSUN2-N domain is shown in cyan, and the NSUN2-C domain is highlighted in red. The rod-like structures illustrate the hydrogen bonds and van der Waals forces within a 3Å radius, indicative of the molecular interactions between these proteins.

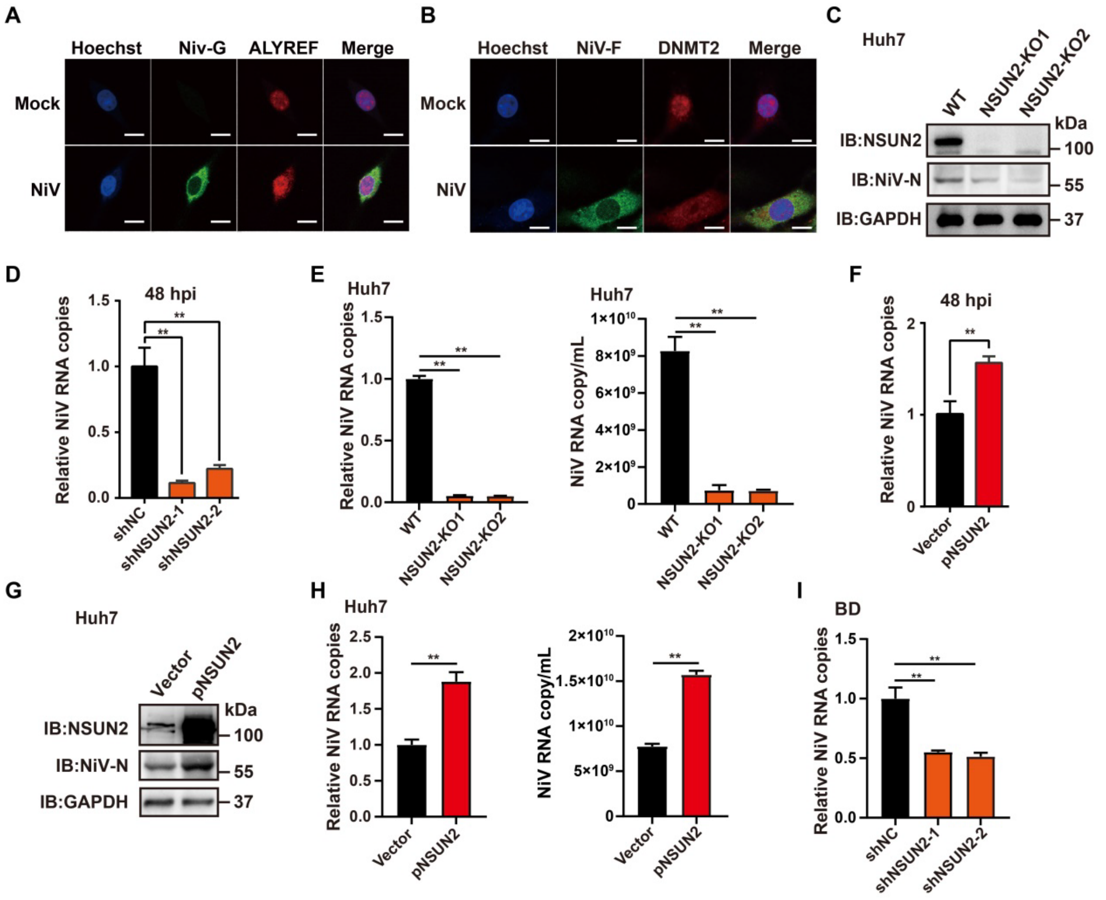

Figure S2. NiV infection reshapes the subcellular localization of m5C-associated proteins and modulates viral replication through NSUN2. (A & B) Confocal microscopy analysis of mock- or NiV-MY-infected Vero cells immunostained with antibodies against NiV-G (green) and ALYREF (red) (A) or against NiV-F (green) and DNMT2 (red) (B). Nuclei were counterstained with Hoechst (blue). Scale bar = 10 μm. (C & G) NSUN2 KO (E) or overexpression (G) Huh7 cells were infected by NiV-MY. NSUN2 and viral protein expression levels were analyzed by western blot. (D, F & I) Vero cells knocked down (D & I) or overexpressing NSUN2 (F) were infected with NiV-MY (D & F) or NiV-BD (I) for 48 hours to measure RNA levels of NiV by qRT-PCR. Data are presented as means ± SEMs (n = 3). ***P* ≤ 0.01, unpaired Student's *t*-tests. (E & H) NiV-MY RNA levels in intracellular fractions or infection supernatants were quantified by qRT-PCR at 48 hpi in Huh7 cells with NSUN2 KO (E) or overexpression (I). Data are presented as means ± SEMs (n = 3). ***P* ≤ 0.01, unpaired Student's *t*-test.

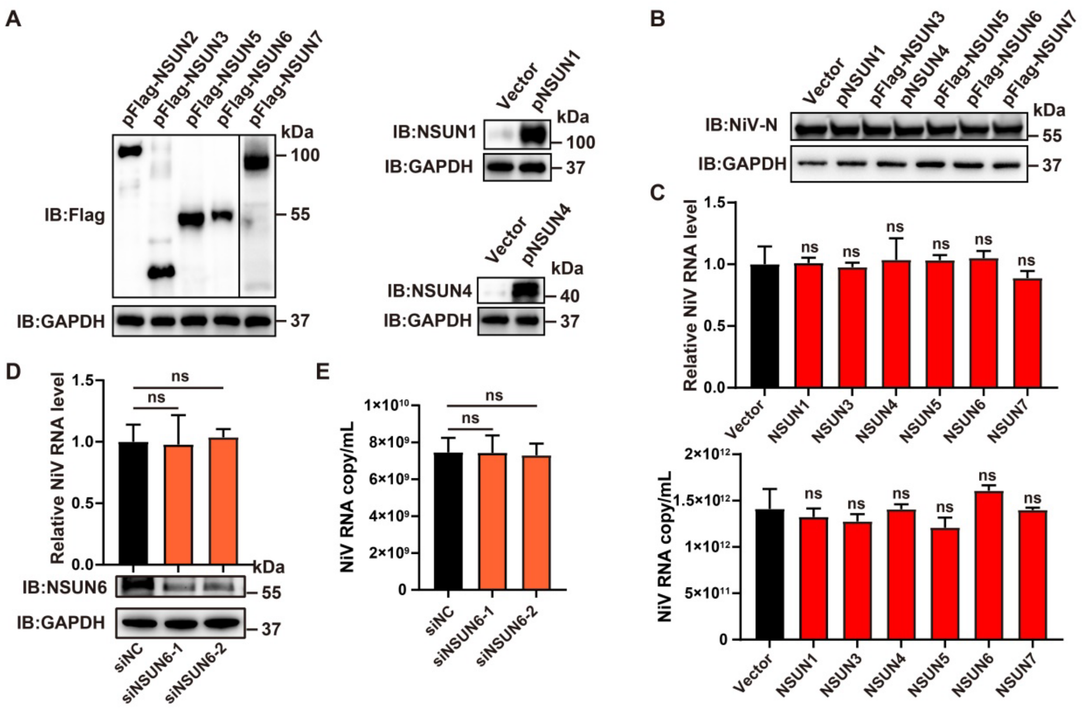

Figure S3. NSUN1 and NSUN3–7 do not affect NiV replication. (A–B) Western blot analysis of NSUN family protein expression (A) and NiV protein levels (B) in Vero cells overexpressing NSUN1 or NSUN3–7. (C) NiV-MY RNA levels in intracellular fractions and culture supernatants from NSUN1- or NSUN3–7–overexpressing Vero cells were quantified by qRT-PCR at 48 hpi. Data are shown as means ± SEMs (n = 3). ns, not significant; unpaired Student's *t*-tests. (D–E) Quantification of NiV-MY RNA levels in the cell lysates (D) and supernatants (E) of NSUN6-knockdown Huh7 cells at 48 hpi. Data are shown as means ± SEMs (n = 3). ns, not significant; unpaired Student's *t*-tests.

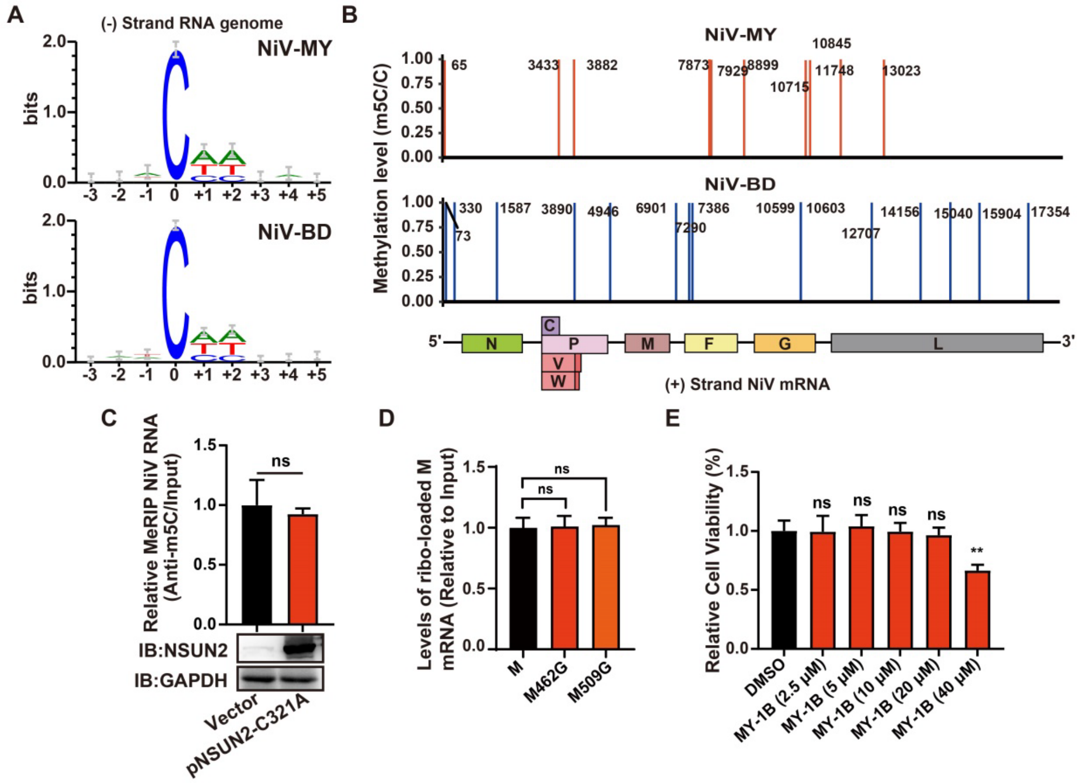

Figure S4. Mapping and functional characterization of m⁵C modifications in NiV RNAs. (A) Conserved sequence of m⁵C in the NiV-MY and NiV-BD genome. (B) Nanopore direct RNA sequencing (DRS) of polyadenylated NiV-MY and NiV-BD mRNAs isolated from infected Vero cells. The vertical axis indicates the per-site m⁵C probability; plotted sites are those with an average probability ≥99%. Data are derived from n = 3 independent experiments. (C) MeRIP-qRT-PCR analysis of m⁵C level in NiV-MY RNA from cells treated with pNSUN2-C321A. Data are represented as means ± SEM (n = 3). ns, not significant, unpaired Student's *t*-test. (D) Ribosome-bound and input M RNA from HEK293T cells transfected with M and mutant RNAs were analyzed using qRT-PCR. Data are presented as means ± SEMs (n = 3). ns: not significant, unpaired Student's *t*-tests. (E) Vero cells were treated with increasing concentrations of MY-1B for 24 h, and cell viability was measured by CCK-8. Data are means ± SEM (n = 3). ns, not significant, unpaired Student's *t*-test.

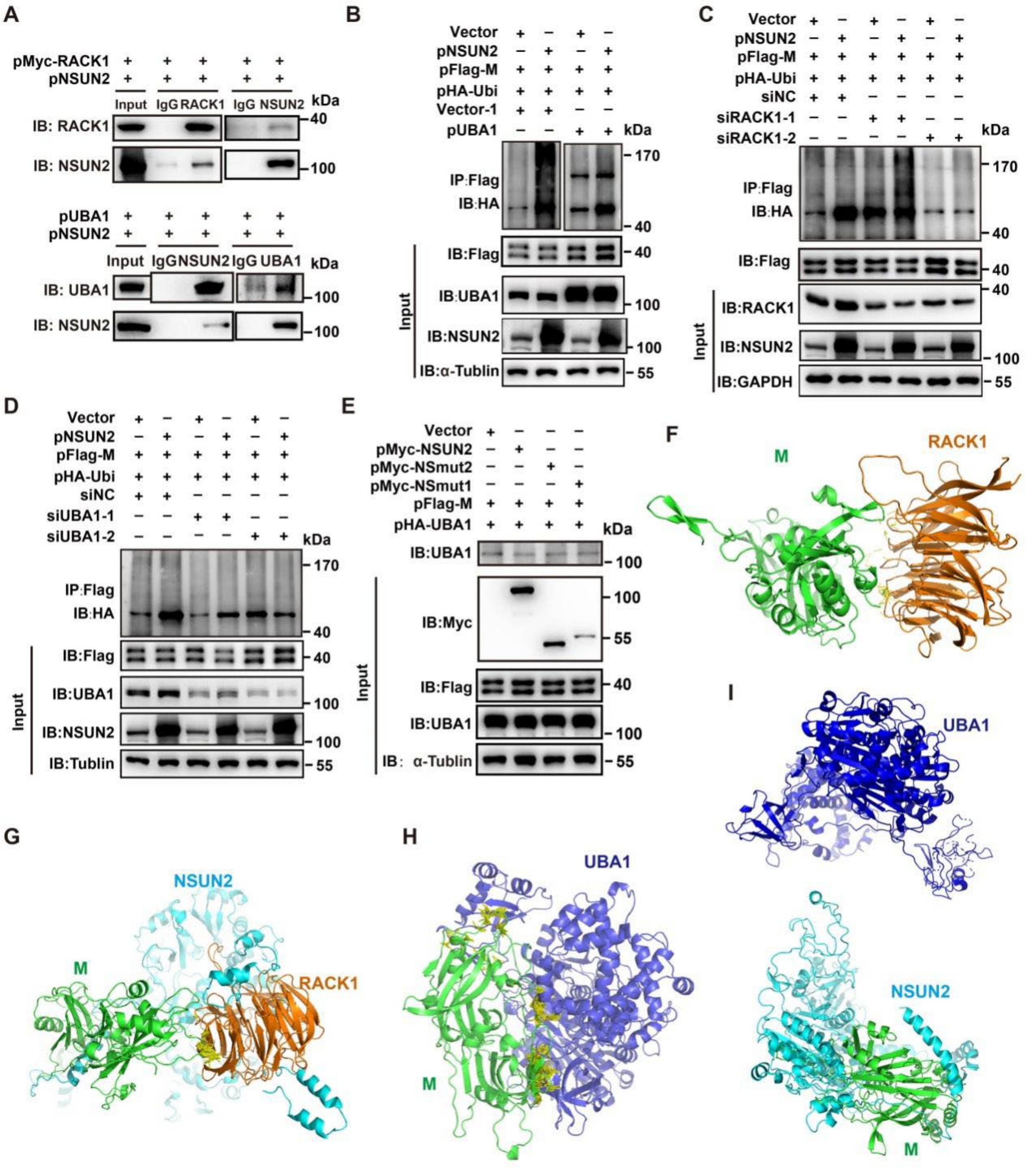

Figure S5. RACK1 and UBA1 participate in M Ubiquitination, but NSUN2 does not enhance their binding to M. (A) HEK293T cells were co-transfected with pNSUN2, pMyc-RACK1, or pUBA1. Co-IP and western blotting was performed to assess interactions between NSUN2 and RACK1 or NSUN2 and UBA1. (B-D) Ubiquitination assays revealed that NSUN2 enhances M protein ubiquitination upon UBA1 overexpression (B), whereas knockdown of RACK1 (C) or UBA1 (D) attenuates NSUN2-mediated M ubiquitination. (E) Co-IP analysis demonstrated the binding of M to UBA1 in HEK293T cells with NSUN2, NSUN2-N, or NSUN2-C overexpression. (F-I) The interactions between M and RACK1 (F) or M and UBA1 (H) in the presence or absence of NSUN2 (G and I) were predicted and visualized by Helixfold3. In the structural models, M protein is depicted as green chains, NSUN2 is shown in cyan, RACK1 is shown in orange, and UBA1 is shown in blue. Rod-like structures represent hydrogen bonds and van der Waals forces within a 3Å radius.

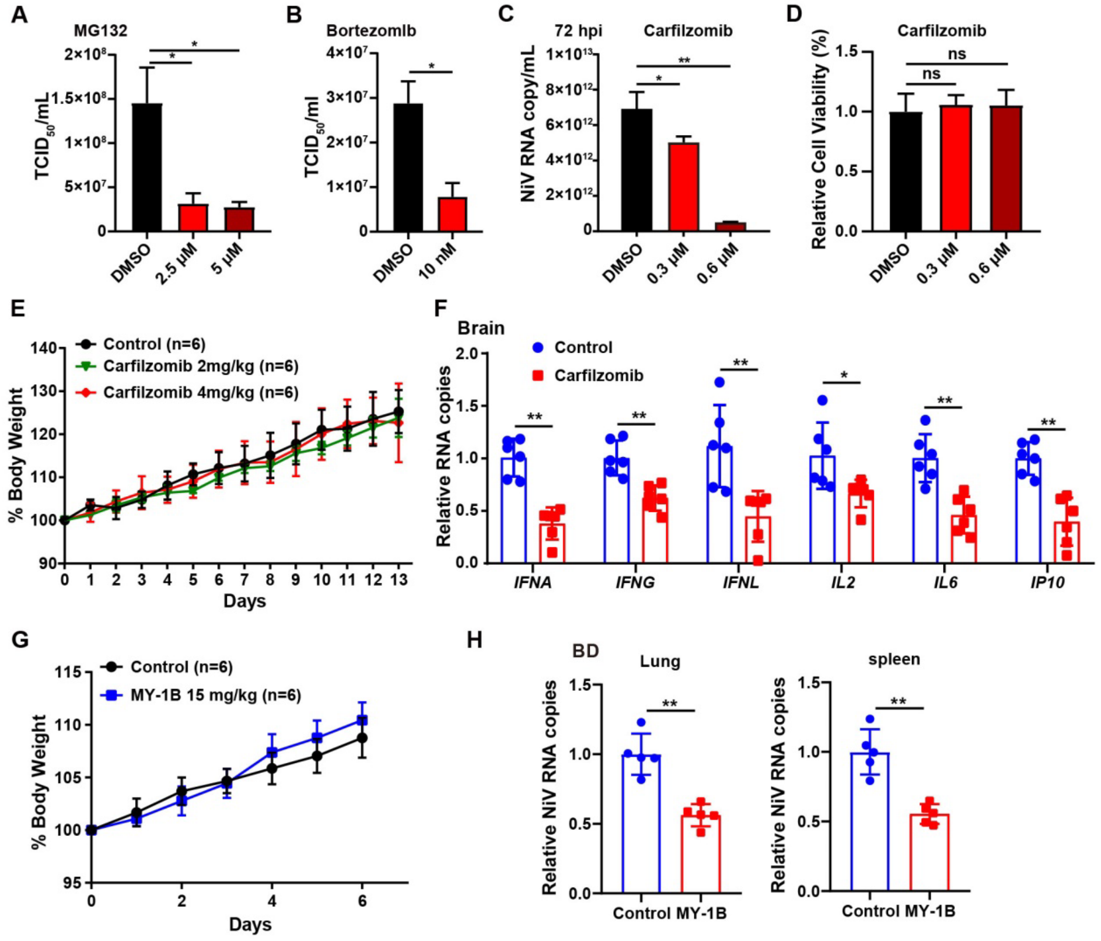

Figure S6. Proteasome inhibitors attenuate viral replication and inflammatory responses. (A & B) Vero cells were pre-treated with DMSO (control), MG132, or bortezomib and subsequently infected with NiV. Viral titers in the supernatant were quantified using the $TCID_{50}$ assay. Data are expressed as means ± SEM (n = 3). Statistical significance was determined using unpaired Student's *t*-tests: **P* ≤ 0.05. (C) Vero cells pre-treated with DMSO or carfilzomib were infected with NiV for 72 hours, and viral RNA copies in the supernatant were measured by qRT-PCR. Data are presented as means ± SEM (n = 3). **P* ≤ 0.05, ***P* ≤ 0.05, unpaired Student's *t*-tests. (D) Vero cells were treated with carfilzomib for 24 h, and cell viability was measured by CCK-8. Data are means ± SEM (n = 3). ns, not significant, unpaired Student's *t*-test. (E & G) Body weight was monitored daily in hamsters treated with control solution, carfilzomib (E), or MY-1B (G) for 13 or 6 days, respectively. (F) Cytokine expression levels in the brains of NiV-infected mice treated with either a control or carfilzomib for 4 days were assessed using qRT-PCR. Data are presented as means ± SEM (n = 3). ***P* ≤ 0.01, unpaired Student's t-tests. (H) qRT-PCR quantification of NiV-BD RNA copies in lung, brain, and spleen tissues of hamsters treated with MY-1B at 4 days post-infection. Data are presented as means ± SEM (n = 3). ***P* ≤ 0.01, unpaired Student's *t*-tests.

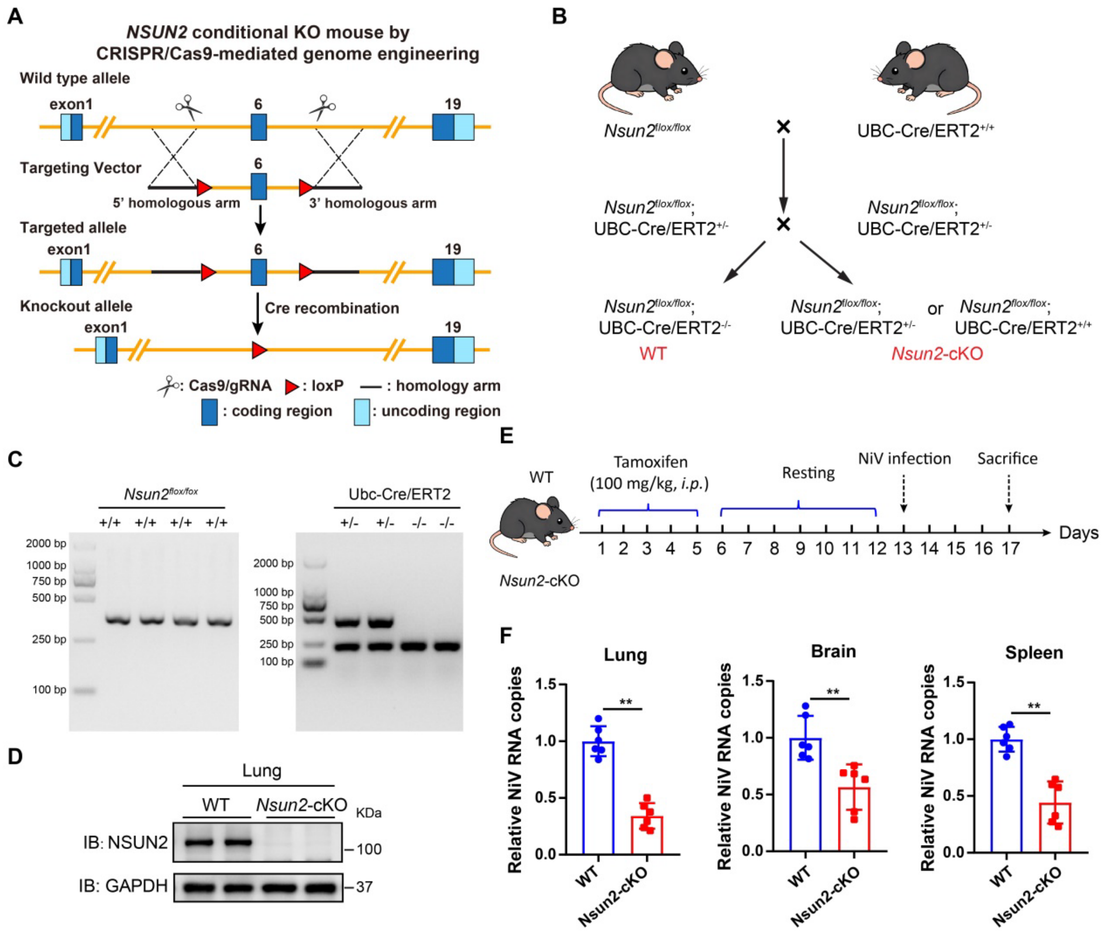

Figure S7. Generation and validation of *Nsun2*-cKO mice and assessment of NiV replication. (A) Exon 6 of the Nsun2 gene was flanked by loxP sites using CRISPR/Cas9 to generate *Nsun2*$^{flox/flox}$ mice. (B) *Nsun2*$^{flox/flox}$ mice were crossed with UBC-Cre/ERT2 mice to obtain *Nsun2*$^{flox/flox}$; UBC-Cre/ERT2$^{-/-}$ (WT) and *Nsun2*$^{flox/flox}$; UBC-Cre/ERT2$^{+/-}$ or *Nsun2*$^{flox/flox}$; UBC-Cre/ERT2$^{+/+}$ (*Nsun2*-cKO) genotype mice. (C & D) Efficient deletion of *Nsun2* was validated by genomic PCR (C) and immunoblotting of Lung tissues (D). (E) WT and *Nsun2*-cKO mice were intraperitoneally injected with tamoxifen (100 mg/kg) for five consecutive days, rested, and subsequently challenged with NiV-MY. Mice were sacrificed at 4 days post-infection. (F) qRT-PCR quantification of NiV-MY RNA levels in lung, brain, and spleen tissues from WT and Nsun2-cKO mice. Data are shown as means ± SEM (n = 3). $^{**}P \leq 0.01$, unpaired Student's *t*-tests.

Table S1. Aipathwell ® Immunohistochemical analysis of M

Names of Images	Positive Area, %	Mean Density	Area Density	H-Score
		**Lung**		
Control-1	46.17%	0.0969	0.044737	90.7691
Control-2	58.84%	0.1196	0.070384	115.0250
Control-3	52.77%	0.1089	0.057494	101.9721
Control-4	55.21%	0.0909	0.050166	114.0410
Control-5	51.27%	0.0889	0.045575	102.1252
Control-6	53.71%	0.0928	0.049858	111.7818
Control-7	46.41%	0.0944	0.043821	91.2826
Control-8	47.06%	0.0848	0.039904	90.9734
Control-9	53.04%	0.1134	0.060127	102.0263
Control-10	50.21%	0.0898	0.045105	101.3421
Control-11	50.15%	0.0832	0.041731	100.0284
Control-12	49.99%	0.0814	0.040679	98.1894
Carfilzomib-1	39.93%	0.0845	0.033746	73.3785
Carfilzomib-2	39.04%	0.0776	0.030295	70.9256
Carfilzomib-3	39.49%	0.0859	0.033927	71.4996
Carfilzomib-4	32.60%	0.0901	0.029379	57.8332
Carfilzomib-5	33.52%	0.0957	0.032074	63.3721
Carfilzomib-6	35.46%	0.0923	0.032746	64.9394
Carfilzomib-7	42.68%	0.1005	0.042913	81.7268
Carfilzomib-8	41.14%	0.0740	0.030432	74.2890
Carfilzomib-9	38.03%	0.0871	0.033121	68.6486
Carfilzomib-10	38.40%	0.0861	0.033053	72.0828
Carfilzomib-11	40.73%	0.0674	0.027453	70.8501
Carfilzomib-12	34.88%	0.0788	0.027481	62.8351
		**Brain**		
Control-1	93.99%	0.0368	0.034576	179.7411
Control-2	89.02%	0.0522	0.046492	169.6867
Control-3	92.98%	0.0440	0.040885	182.0153
Control-4	94.62%	0.0449	0.042525	185.3040
Control-5	88.01%	0.0526	0.046318	164.7189
Control-6	87.99%	0.0496	0.043607	160.3446
Control-7	88.70%	0.0403	0.035766	171.2216
Control-8	88.18%	0.0598	0.052771	170.5487
Control-9	86.46%	0.0592	0.051143	168.5160
Control-10	88.57%	0.0538	0.047630	167.2703
Control-11	92.41%	0.0524	0.048451	178.2751
Control-12	92.78%	0.0369	0.034233	177.5041
Carfilzomib-1	40.28%	0.0414	0.016678	66.1753
Carfilzomib-2	45.95%	0.0399	0.018341	76.7364

Carfilzomib-3	53.64%	0.0431	0.023113	93.6810
Carfilzomib-4	62.25%	0.0540	0.033645	109.7271
Carfilzomib-5	46.47%	0.0627	0.029142	87.5612
Carfilzomib-6	44.30%	0.0548	0.024262	74.8998
Carfilzomib-7	49.51%	0.0566	0.028002	87.2950
Carfilzomib-8	53.65%	0.0340	0.018249	92.2691
Carfilzomib-9	52.69%	0.0472	0.024871	89.5329
Carfilzomib-10	55.97%	0.0293	0.016385	100.8213
Carfilzomib-11	56.15%	0.0380	0.021323	102.0341
Carfilzomib-12	56.65%	0.0480	0.027198	100.8533
**Spleen**				
Control-1	36.96%	0.0670	0.024752	61.3283
Control-2	41.38%	0.0609	0.025218	68.0679
Control-3	37.04%	0.0639	0.023660	61.2379
Control-4	33.06%	0.0640	0.021148	49.5121
Control-5	32.22%	0.0639	0.020582	49.0325
Control-6	43.08%	0.0668	0.028790	73.5992
Control-7	43.56%	0.0704	0.030671	75.2194
Carfilzomib-1	8.65%	0.0632	0.005465	10.8303
Carfilzomib-2	13.94%	0.0635	0.008854	18.9455
Carfilzomib-3	11.99%	0.0938	0.011253	18.4891
Carfilzomib-4	28.28%	0.0663	0.018761	41.4765
Carfilzomib-5	25.13%	0.0713	0.017923	36.6929
Carfilzomib-6	28.09%	0.0627	0.017621	40.7159
Carfilzomib-7	26.32%	0.0650	0.017094	36.9530

Table S2. Pathological Score of the Lungs in NiV-Infected Hamsters

Lesion / No	Alveolar Wall Thickening	Inflammatory Cell Infiltration	Necrosis	Hemorrhage	Total Score
Control-1	1	1	0	2	4
Control-2	0	0	0	0	0
Control-3	2	1	1	2	6
Control-4	2	1	1	2	6
Control-5	3	1	1	1	6
Control-6	2	1	1	2	6
Carfilzomib-1	0	0	0	0	0
Carfilzomib-2	0	1	0	1	2
Carfilzomib-3	0	0	0	0	0
Carfilzomib-4	0	0	0	0	0
Carfilzomib-5	0	1	0	1	2
Carfilzomib-6	0	1	0	0	1

Table S3. Pathological Score of the Spleen in NiV-Infected Hamsters

No \ Lesion	Inflammatory Cell Infiltration	Necrosis	Congestion	Total Score
Control-1	0	1	1	2
Control-2	1	2	0	3
Control-3	1	1	1	3
Carfilzomib-1	0	0	0	0
Carfilzomib-2	0	0	0	0
Carfilzomib-3	1	0	0	1

Table S4. Pathological Score of the Brain in NiV-Infected Hamsters

No \ Lesion	Neuron Shrinkage	Neuron Degeneration	Congestion	Glial Cell Proliferation	Total Score
Control-1	1	1	0	0	2
Control-2	3	1	1	0	5
Control-3	2	1	1	0	4
Control-4	2	1	0	0	3
Control-5	3	0	1	0	4
Control-6	1	2	2	0	5
Carfilzomib-1	0	2	0	0	2
Carfilzomib-2	0	2	0	0	2
Carfilzomib-3	1	1	2	0	4
Carfilzomib-4	1	1	2	0	4
Carfilzomib-5	1	1	1	0	3
Carfilzomib-6	0	1	2	0	3
